# Comparative genomic and phylogenomic analyses of the *Bifidobacteriaceae* family

**DOI:** 10.1186/s12864-017-3955-4

**Published:** 2017-08-01

**Authors:** Gabriele Andrea Lugli, Christian Milani, Francesca Turroni, Sabrina Duranti, Leonardo Mancabelli, Marta Mangifesta, Chiara Ferrario, Monica Modesto, Paola Mattarelli, Killer Jiří, Douwe van Sinderen, Marco Ventura

**Affiliations:** 1 Laboratory of Probiogenomics, Department of Chemistry, Life Sciences and Environmental Sustainability, University of Parma, Parma, Italy; 2 GenProbio srl, Parma, Italy; 3 Department of Agricultural Sciences, University of Bologna, Bologna, Italy; 4 Department of Microbiology, Nutrition and Dietetics, Faculty of Agrobiology, Food and Natural Resources, Czech University of Life Sciences Prague, Prague, Czech Republic; 5 Institute of Animal Physiology and Genetics v.v.i., Academy of Sciences of the Czech Republic, Prague, Czech Republic; 6 APC Microbiome Institute and School of Microbiology, National University of Ireland, Cork, Ireland

**Keywords:** *Bifidobacteriaceae*, Genomics, Phylogenomics, *Bifidobacterium*, Bifidobacteria

## Abstract

**Background:**

Members of the *Bifidobacteriaceae* family represent both dominant microbial groups that colonize the gut of various animals, especially during the suckling stage of their life, while they also occur as pathogenic bacteria of the urogenital tract. The pan-genome of the genus *Bifidobacterium* has been explored in detail in recent years, though genomics of the *Bifidobacteriaceae* family has not yet received much attention. Here, a comparative genomic analyses of 67 *Bifidobacteriaceae* (sub) species including all currently recognized genera of this family, i.e., *Aeriscardovia*, *Alloscardovia*, *Bifidobacterium*, *Bombiscardovia*, *Gardnerella*, *Neoscardovia*, *Parascardovia*, *Pseudoscardovia* and *Scardovia,* was performed. Furthermore, in order to include a representative of each of the 67 (currently recognized) (sub) species belonging to the *Bifidobacteriaceae* family, we sequenced the genomes of an additional 11 species from this family, accomplishing the most extensive comparative genomic analysis performed within this family so far.

**Results:**

Phylogenomics-based analyses revealed the deduced evolutionary pathway followed by each member of the *Bifidobacteriaceae* family, highlighting *Aeriscardovia aeriphila* LMG 21773 as the deepest branch in the evolutionary tree of this family. Furthermore, functional analyses based on genome content unveil connections between a given member of the family, its carbohydrate utilization abilities and its corresponding host. In this context, bifidobacterial (sub) species isolated from humans and monkeys possess the highest relative number of acquired glycosyl hydrolase-encoding genes, probably in order to enhance their metabolic ability to utilize different carbon sources consumed by the host.

**Conclusions:**

Within the *Bifidobacteriaceae* family, genomics of the genus *Bifidobacterium* has been extensively investigated. In contrast, very little is known about the genomics of members of the other eight genera of this family. In this study, we decoded the genome sequences of each member of the *Bifidobacteriaceae* family. Thanks to subsequent comparative genomic and phylogenetic analyses, the deduced pan-genome of this family, as well as the predicted evolutionary development of each taxon belonging to this family was assessed.

**Electronic supplementary material:**

The online version of this article (doi:10.1186/s12864-017-3955-4) contains supplementary material, which is available to authorized users.

## Background

The *Bifidobacteriaceae* is the sole family member of the *Bifidobacteriales* order, and has been shown to represent the deepest branch within the *Actinobacteria* phylum [1]. Currently, the *Bifidobacteriaceae* family includes 55 (sub) species of the genus *Bifidobacterium* [1, 2] and members of eight additional genera, i.e., *Aeriscardovia*, *Alloscardovia*, *Bombiscardovia*, *Gardnerella*, *Neoscardovia*, *Parascardovia*, *Pseudoscardovia* and *Scardovia*, which together encompass 12 species [1, 3]. Furthermore, a novel, yet unculturable species was identified from termites and included in the *Bifidobacteriaceae* family with the taxonomic denomination of ‘*Candidatus* Ancillula trichonymphae’ [4]. The name of this latter organism originates from a variety of flagellates of the genus *Trichonympha*, of which this strain is symbiont [4].

*Bifidobacteriaceae* are chemoorganotrophs with a fermentative type of metabolism, Gram-positive, non-spore-forming, non-motile, and anaerobic or facultative anaerobic bacteria [5]. They reside in different ecological niches, such as the human and animal gastrointestinal tract (GIT), oral cavity and the (social) insect gut [6], while they may also be found in blood and sewage, possibly due to environmental contamination. Many bifidobacteria are appreciated for their purported health-promoting activities as well as their relevance in early life colonization and contributions to the infant gut glycobiome [7]. Conversely, members of the other genera of the *Bifidobacteriaceae* family are generally associated with human and animal dental caries, and are commonly isolated from human clinical samples of tonsil abscesses and bacterial vaginosis [8, 9]. Furthermore, in contrast to bifidobacteria, which mainly include strict anaerobes with some exceptions, such as *Bifidobacterium animalis* subsp. *lactis* and *Bifidobacterium asteroides* [10, 11], other members of the family can grow under aerobic conditions and possess DNA with a lower G + C content [12–14].

The most controversial species belonging to this family is *Gardnerella vaginalis* [9], originally described by Leopold in 1953 and named *Haemophilus vaginalis* [15]. Subsequently, taxonomic studies mixed with data obtained from biochemical analyses and electron microscopic examinations, supported the need for its re-classification as a new genus [16, 17]. Currently, *G. vaginalis* is described as an opportunistic pathogen whose presence is tightly associated with bacterial vaginosis [18, 19]. Furthermore, *Parascardovia denticolens* and *Scardovia inopinata*, which were classified in 1996 as *Bifidobacterium denticolens* and *Bifidobacterium inopinatum* [20], respectively, together with *Scardovia wiggsiae* and *Bifidobacterium dentium* are associated with human dental caries [21]. While members of these species are present at high numbers in the saliva of adults, their presence strongly correlates with other caries-associated organisms [22, 23].

Notably, comparative genome analyses of the genus *Bifidobacterium* have been targeting the entire genus [2, 24] or one specific bifidobacterial taxa, i.e., *B. bifidum* [25], *B. adolescentis* [26], *B. breve* [27], *B. longum* [28] or the *B. animalis* subsp. *lactis* taxon [11]. In contrast, the genomics of the other eight genera belonging to the *Bifidobacteriaceae* family have not yet been investigated in detail. Here, we decoded the genomes of 11 species belonging to the *Bifidobacteriaceae* family for which there was no prior genomic data. Furthermore, we performed an in depth comparative genomic analysis, as well as a phylogenetic reconstruction of the 67 (sub) species currently assigned to the *Bifidobacteriaceae* family.

## Methods

### *Bifidobacteriaceae* strains

We retrieved the complete and partial genome sequences of 56 *Bifidobacteriaceae* strains from the National Center for Biotechnology Information (NCBI) public database (Table 1). Additionally, we sequenced and analyzed the genome sequences of 11 *Bifidobacteriaceae* strains deposited in the GenBank sequence database (Table 2).

**Table 1:** General features of *Bifidobacteriaceae* genomes

Taxon number	*Bifidobacteriaceae* strains	Genome status^a^	Genome size	GC content	ORFs number	rRNA loci	tRNA number	GHs number	GH index	Isolation	Accession number
01	*B. actinocoloniiforme* DSM 22766	Draft (4)	1,823,388	62.71	1484	2	46	41	0.0276	Bumblebee digestive tract	JGYK00000000
02	*B. adolescentis* ATCC 15703	Complete	2,089,645	59.18	1649	5	54	81	0.0491	Intestine of adult	AP009256.1
03	*B. aesculapii* DSM 26737	Draft (118)	2,794,396	64.58	2172	6	60	82	0.0378	Faeces of baby common marmosets	BCFK00000000
04	*B. angulatum* LMG 11039	Draft (6)	2,003,806	59.41	1523	4	48	63	0.0414	Human faeces	JGYL00000000
05	*B. animalis* subsp. *animalis* LMG 10508	Draft (13)	1,915,007	60.47	1527	3	52	48	0.0314	Rat feces	JGYM00000000
06	*B. animalis* subsp. *lactis* DSM 10140	Complete	1,938,606	60.48	1518	4	52	53	0.0349	Fermented milk	CP001606.1
07	*B. aquikefiri* LMG 28769	Draft (18)	2,408,364	52.29	2000	2	45	56	0.0280	Household water kefir	MWXA00000000
08	*B. asteroides* LMG 10735 (PRL2011)	Complete	2,167,304	60.05	1653	2	44	58	0.0351	Honeybee hindgut	CP003325.1
09	*B. biavatii* DSM 23969	Draft (56)	3,252,147	63.1	2557	5	61	137	0.0536	Feces of tamarin	JGYN00000000
10	*B. bifidum* LMG 11041	Draft (2)	2,208,468	62.67	1704	3	53	66	0.0387	Brest-feed Infant feaces	JGYO00000000
11	*B. bohemicum* DSM 22767	Draft (5)	2,052,470	57.45	1632	2	47	56	0.0343	Bumblebee digestive tract	JGYP00000000
12	*B. bombi* DSM 19703	Draft (4)	1,895,239	56.08	1454	2	48	44	0.0303	Bumblebee digestive tract	ATLK00000000
13	*B. boum* LMG 10736	Draft (18)	2,171,356	59.31	1726	4	49	45	0.0261	Bovine rumen	JGYQ00000000
14	*B. breve* LMG 13208	Draft (31)	2,263,780	58.88	1887	2	53	67	0.0355	Infant intestine	JGYR00000000
15	*B. callitrichos* DSM 23973	Draft (33)	2,887,313	63.52	2364	3	58	105	0.0444	Feces of common marmoset	JGYS00000000
16	*B. catenulatum* LMG 11043	Draft (11)	2,082,756	56.11	1664	5	55	91	0.0547	Adult intestine	JGYT00000000
17	*B. choerinum* LMG 10510	Draft (20)	2,096,123	65.53	1672	3	55	54	0.0323	Piglet faeces	JGYU00000000
18	*B. commune* R-52791	Dreaft (4)	1,633,662	53.93	1303	1	47	31	0.0238	Bumble bee gut	FMBL00000000
19	*B. coryneforme* LMG 18911	Complete	1,755,151	60.51	1364	3	56	43	0.0315	Honeybee hindgut	CP007287
20	*B. crudilactis* LMG 23609	Draft (6)	2,362,816	57.72	1883	2	45	51	0.0271	Raw cow milk	JHAL00000000
21	*B. cuniculi* LMG 10738	Draft (41)	2,531,592	64.87	2194	4	63	70	0.0319	Rabbit faeces	JGYV00000000
22	*B. dentium* LMG 11045 (Bd1)	Complete	2,636,367	58.54	2129	4	55	113	0.0531	Oral cavity	CP001750.1
23	*B. eulemuris* DSM 100216	Draft (34)	2,913,389	62.2	2331	2	53	126	0.0541	Faeces of the black lemur	MWWZ00000000
24	*B. gallicum* LMG 11596	Draft (12)	2,004,594	57.61	1507	2	58	45	0.0299	Adult intestine	JGYW00000000
25	*B. gallinarum* LMG 11586	Draft (10)	2,160,836	64.22	1654	2	53	78	0.0472	Chicken caecum	JGYX00000000
26	*B. hapali* DSM 100202	Draft (76)	2,834,308	54.5	2253	3	54	121	0.0537	Faeces of baby common marmosets	MWWY00000000
27	*B. indicum* LMG 11587	Complete	1,734,546	60.49	1352	3	47	41	0.0303	Insect	CP006018
28	*B. kashiwanohense* DSM 21854	Draft (30)	2,307,960	56.2	1948	5	53	91	0.0467	Infant feaces	JGYY00000000
29	*B. lemurum* DSM 28807	Draft (38)	2,944,293	62.64	2321	3	49	122	0.0526	Faeces of the ring-tailed lemur	MWWX00000000
30	*B. longum* subsp. *infantis* ATCC 15697	Complete	2,832,748	59.86	2500	4	79	71	0.0284	Intestine of infant	AP010889.1
31	*B. longum* subsp. *longum* LMG 13197	Draft (8)	2,384,703	60.33	1899	3	71	73	0.0384	Adult intestine	JGYZ00000000
32	*B. longum* subsp. *suis* LMG 21814	Draft (36)	2,335,832	59.96	1955	3	55	74	0.0379	Pig faeces	JGZA00000000
33	*B. magnum* LMG 11591	Draft (13)	1,822,476	58.72	1507	5	56	46	0.0305	Rabbit faeces	JGZB00000000
34	*B. merycicum* LMG 11341	Draft (16)	2,280,236	60.33	1741	3	53	66	0.0379	Bovine rumen	JGZC00000000
35	*B. minimum* LMG 11592	Draft (18)	1,892,860	62.73	1590	2	53	41	0.0258	Sewage	JGZD00000000
36	*B. mongoliense* DSM 21395	Draft (43)	2,170,490	62.78	1798	2	47	65	0.0362	Fermented mare’s milk	JGZE00000000
37	*B. moukalabense* DSM 27321	Draft (12)	2,515,335	59.87	2046	4	56	105	0.0513	Feces of wild western lowland gorilla	AZMV00000000
38	*B. myosotis* DSM 100196	Draft (58)	2,944,195	62.55	2168	4	56	101	0.0466	Faeces of baby common marmosets	MWWW00000000
39	*B. pseudocatenulatum* LMG 10505	Draft (10)	2,283,767	56.36	1771	6	53	85	0.0480	Infant faeces	JGZF00000000
40	*B. pseudolongum* subsp. *globosum* LMG 11569	Draft (26)	1,935,255	63.39	1574	4	52	53	0.0337	Bovine rumen	JGZG00000000
41	*B. pseudolongum* subsp. *pseudolongum* LMG 11571	Draft (11)	1,898,684	63.06	1495	3	52	57	0.0381	Swine faeces	JGZH00000000
42	*B. psychraerophilum* LMG 21775	Draft (11)	2,615,078	58.75	2122	1	45	80	0.0377	Pig caecum	JGZI00000000
43	*B. pullorum* DSM 20433	Draft (38)	2,100,948	64.31	1678	2	51	81	0.0479	Faeces of chicken	JDUI00000000
44	*B. reuteri* DSM 23975	Draft (28)	2,847,572	60.45	2149	4	53	85	0.0396	Feces of common marmoset	JGZK00000000
45	*B. ruminantium* LMG 21811	Draft (23)	2,249,807	59.18	1832	4	50	62	0.0338	Bovine rumen	JGZL00000000
46	*B. saeculare* LMG 14934	Draft (14)	2,263,283	63.75	1857	2	48	82	0.0442	Rabbit faeces	JGZM00000000
47	*B. saguini* DSM 23967	Draft (33)	2,787,036	56.35	2321	5	59	104	0.0448	Feces of tamarin	JGZN00000000
48	*B. scardovii* LMG 21589	Draft (34)	3,141,793	64.63	2480	3	55	128	0.0516	Blood	JGZO00000000
49	*B. stellenboschense* DSM 23968	Draft (40)	2,812,864	65.34	2202	6	59	80	0.0363	Feces of tamarin	JGZP00000000
50	*B. subtile* LMG 11597	Draft (27)	2,790,088	60.92	2260	1	47	56	0.0248	Sewage	JGZR00000000
51	*B. thermacidophilum* subsp. *porcinum* LMG 21689	Draft (3)	2,079,368	60.2	1738	3	40	46	0.0265	Piglet faeces	JGZS00000000
52	*B. thermacidophilum* subsp. *thermacidophilum* LMG 21395	Draft (8)	2,233,072	60.38	1823	4	48	47	0.0258	Anaerobic digester	JGZT00000000
53	*B. thermophilum* DSM 20212	Draft (50)	2,252,351	60.07	1756	3	49	58	0.0341	Bovine rumen	JHWM00000000
54	*B. tissieri* DSM 100201	Draft (38)	2,873,483	61.05	2260	2	60	79	0.0350	Faeces of baby common marmosets	MWWV00000000
55	*B. tsurumiense* JCM 13495	Draft (25)	2,164,426	52.84	1629	3	46	85	0.0522	Hamster dental plaque	JGZU00000000
56	*Aeriscardovia aeriphila* LMG 21773	Draft (12)	1,631,097	54.03	1288	3	47	53	0.0411	Pig caecum	MWWU00000000
57	*Alloscardovia criceti* DSM 17774	Draft (11)	1,884,654	50.06	1524	4	45	58	0.0381	Dental plaque, golden hamster	AQXR00000000
58	*Alloscardovia macacae* DSM 24762	Draft (20)	1,891,581	55.82	1552	4	48	50	0.0322	Milk of a female macaque bred	MWWT00000000
59	*Alloscardovia omnicolens* DSM 21503	Draft (43)	1,847,146	46.65	1564	3	47	56	0.0358	Human tonsil	ATVB00000000
60	*Bombiscardovia coagulans* DSM 22924	Draft (15)	1,741,326	47.33	1441	2	45	36	0.0250	Bumblebee digestive tract	MWWS00000000
61	*Gardnerella vaginalis* ATCC 14018	Complete	1,667,406	41.36	1271	2	45	41	0.0323	Vaginal secretions	AP012332
62	*Neoscardovia arbecensis* 1879	Draft (21)	1,971,875	54.25	1641	2	45	56	0.0341	Feces of rabbit	n.d.
63	*Parascardovia denticolens* DSM 10105	Complete	1,890,857	58.31	1528	2	45	56	0.0366	Human dental caries	AP012333
64	*Pseudoscardovia radai* DSM 24742	Draft (35)	2,436,770	65.03	1779	3	47	41	0.0230	Digestive tract of wild pig *Sus scrofa*	MWWR00000000
65	*Pseudoscardovia suis* DSM 24744	Draft (26)	2,270,618	60.57	1736	3	48	45	0.0259	Digestive tract of wild pig Sus scrofa	MWWQ00000000
66	*Scardovia inopinata* JCM 12537	Complete	1,797,862	48.63	1465	2	46	49	0.0334	Human dental caries	AP012334
67	*Scardovia wiggsiae* F0424	Complete	1,550,817	52.93	1244	2	45	32	0.0257	Human dental caries	AKCI00000000

^a^Numbers in brackets indicate the numbers of assembled contigs

**Table 2:** Sequencing data of the *Bifidobacteriaceae* genomes

*Bifidobacteriaceae* Strains	Genome status^a^	Sequence coverage	Genome size	GC content	ORFs number	rRNA loci	tRNA number	Isolation	Accession number
*Aeriscardovia aeriphila* LMG 21773	Draft (12)	259.66	1,631,097	54.03	1288	3	47	Pig caecum	MWWU00000000
*Alloscardovia macacae* DSM 24762	Draft (20)	173.37	1,891,581	55.82	1552	4	48	Milk of a female macaque bred	MWWT00000000
*Bifidobacterium aquikefiri* LMG 28769	Draft (18)	96.25	2,408,364	52.29	2000	2	45	Household water kefir	MWXA00000000
*Bifidobacterium eulemuris* DSM 100216	Draft (34)	75.23	2,913,389	62.2	2331	2	53	Faeces of the black lemur	MWWZ00000000
*Bifidobacterium hapali* DSM 100202	Draft (76)	109.83	2,834,308	54.5	2253	3	54	Faeces of baby common marmosets	MWWY00000000
*Bifidobacterium lemurum* DSM 28807	Draft (38)	71.87	2,944,293	62.64	2321	3	49	Faeces of the ring-tailed lemur	MWWX00000000
*Bifidobacterium myosotis* DSM 100196	Draft (58)	57.03	2,944,195	62.55	2168	4	56	Faeces of baby common marmosets	MWWW00000000
*Bifidobacterium tissieri* DSM 100201	Draft (38)	66.77	2,873,483	61.05	2260	2	60	Faeces of baby common marmosets	MWWV00000000
*Bombiscardovia coagulans* DSM 22924	Draft (15)	220.35	1,741,326	47.33	1441	2	45	Bumblebee digestive tract	MWWS00000000
*Pseudoscardovia radai* DSM 24742	Draft (35)	145.91	2,436,770	65.03	1779	3	47	Digestive tract of wild pig Sus scrofa	MWWR00000000
*Pseudoscardovia suis* DSM 24744	Draft (26)	140.27	2,270,618	60.57	1736	3	48	Digestive tract of wild pig Sus scrofa	MWWQ00000000

^a^Numbers in brackets indicate the numbers of assembled contigs

### Bacterial strains and growth condition

*Bifidobacteriaceae* pure cultures were inoculated in de Man-Rogosa-Sharpe (MRS) medium (Scharlau Chemie) supplemented with 0.05% (wt/vol) L-cysteine hydrochloride and were grown in an anaerobic atmosphere (2.99% H_2_, 17.01% CO_2_, and 80% N_2_) in a chamber (Concept 400, Ruskin) at 37 °C for 16 h. DNA was extracted as described previously [29] and subjected to further phenol-chloroform purification using a previously described protocol [30].

### Genome sequencing and assemblies

DNA extracted from the various *Bifidobacteriaceae* strains was subjected to whole genome sequencing using MiSeq (Illumina, UK) at GenProbio srl (Parma, Italy) following the supplier’s protocol (Illumina, UK). Fastq files of the paired-end reads obtained from targeted genome sequencing of the isolated strains were used as input for genome assemblies through the MEGAnnotator pipeline [31]. The MIRA program (version 4.0.2) was used for de novo assembly of each *Bifidobacteriaceae* genome sequence [32].

### Sequence annotation

Protein-encoding open reading frames (ORFs) were predicted using Prodigal [33]. Transfer RNA genes were identified using tRNAscan-SE v1.4 [34], while ribosomal RNA genes were detected using RNAmmer v1.2 [35]. Results of the gene-finder program were combined with data from RAPSearch2 analysis (Reduced Alphabet based Protein similarity Search) [36] of a non-redundant protein database provided by the National Center for Biotechnology Information (NCBI) and Hidden Markov Model profile (HMM) search (http://hmmer.org/) in the manually curated Pfam-A protein family database [37]. The combined results were inspected by Artemis [38], which was used for manual editing purposes aimed at verifying and, where necessary, redefining the start of each predicted coding region, and to remove or add coding regions.

### *Bifidobacteriaceae* pan-genome analysis

For the 67 genome sequences of each member of the *Bifidobacteriaceae* family, a pan-genome calculation was performed using the PGAP pipeline [39]. The ORF contents from all genomes used in this study were organized in functional clusters using the GF (Gene Family) method involving comparison of each protein to all other proteins using BLAST analysis (cutoff E-value of 1 X 10^−5^ and 50% identity over at least 50% of both protein sequences), followed by clustering into protein families, named *Bifidobacteriaceae*-specific clusters of orthologous groups (BaeCOGs), using MCL (graph-theory-based Markov clustering algorithm) [40]. A pan-genome profile was built using an optimized algorithm incorporated in PGAP software, based on a presence/absence matrix that included all identified BaeCOGs in the analyzed genomes. Following this, unique protein families for each of the 67 *Bifidobacteriaceae* genomes were classified. Protein families shared between all genomes, named core BaeCOGs, were defined by selecting the families that contained at least one protein member for each genome.

### Phylogenetic comparison

The concatenated core genome sequence of the family (core BaeCOGs)*,* was aligned using MAFFT [41], and phylogenetic trees were constructed using the neighbor-joining method in Clustal W, version 2.1 [42]. The core genome supertree was built using FigTree (http://tree.bio.ed.ac.uk/software/figtree/). Values of average nucleotide identity (ANI) were calculated using the program JSpecies, version 1.2.1 [43].

### Functional analysis

The prediction of genes encoding enzymes that possess structurally-related catalytic and carbohydrate-binding modules catalyzing hydrolysis, modification or synthesis of glycoside bounds was performed by means of the CAZy database [44]. Functional annotation of each gene was performed employing the eggNOG database [45]. A survey of complete pathways involved in both primary and secondary metabolism was performed by means of the MetaCyc metabolic pathways database [46]. Gene function was predicted using a cutoff E-value of 1 × 10^−10^ to identify the best hit from each database.

### Gene gain/loss through evolution reconstruction

Predicting gene acquisition or gene loss as a result of evolution of the bacterial species with at least four available genomes was performed with Count software [47] using Dollo’s parsimony.

## Results and discussion

### General genome features of *Bifidobacteriaceae* genomes

Genome sequences of six bifidobacterial species, i.e., *Bifidobacterium aquikefiri* LMG 28769*, Bifidobacterium eulemuris* DSM 100216, *Bifidobacterium hapali* DSM 100202, *Bifidobacterium lemurum* DSM 28807, *Bifidobacterium myosotis* DSM 100196 and *Bifidobacterium tissieri* DSM 100201, as well as five chromosomes belonging to different genera of the *Bifidobacteriaceae* family, including *Aeriscardovia aeriphila* LMG 21773, *Alloscardovia macacae* DSM 24762, *Bombiscardovia coagulans* DSM 22924 and *Pseudoscardovia radai* DSM 24742 and *Pseudoscardovia suis* DSM 24744, were decoded through shotgun sequencing. Genome features and sequencing data of these 11 *Bifidobacteriaceae* genomes are summarized in Table 2. In order to provide a complete genome analysis of the *Bifidobacteriaceae* family, a representative genome sequence for each of the currently described 67 (sub) species belonging to this family, was retrieved from the NCBI public database (Table 1). Due to the incomplete genome sequences of *Candidatus* Ancillula trichonymphae ImTpAt recovered from the NCBI database, and the impossibility to retrieve this strain from any public bacterial culture collection, we decided to exclude the genome sequences of ImTpAt from our analyses. The combination of genomic data of 56 previously characterized bifidobacterial taxa [2, 3, 48–51] with the chromosome sequences of the 11 *Bifidobacteriaceae* species reported here, resulted in the most comprehensive database of genome sequences of representative members of the *Bifidobacteriaceae* family. The *Bifidobacteriaceae* genomes have an average genome length of 2.25 Mb, and range in size from 1.55 Mb for *Scardovia wiggsiae* F0424 to 3.25 Mb for *Bifidobacterium biavatii* DSM 23969, corresponding to 1244 and 2557 predicted protein-encoding open reading frames (ORFs), respectively (Table 1). Average genomic GC content ranges from 41.36% for *Gardnerella vaginalis* ATCC 14018 to 65.53% for *Bifidobacterium choerinum* LMG 10510, and revealed a higher average for the bifidobacterial strains (60.24%) as compared to the other taxa of the *Bifidobacteriaceae* genera (52.91%) [52]. The average genome size of bifidobacterial strains is also higher than that observed for the other family members, being 2.33 Mb and 1.88 Mb, respectively, highlighting a gene ratio of 1.24 (obtained by dividing the average gene number of bifidobacterial (sub) species with that of the non-bifidobacterial taxa of the *Bifidobacteriaceae* family) in favor of the bifidobacterial strains. The larger gene complement possessed by members of the *Bifidobacterium* genus may reflect an increased genetic variability of this genus, endowing bifidobacteria with an enhanced ability to adapt to a broad range of ecological niches as compared to other members of the *Bifidobacteriaceae* family, which, as is listed in Table 1, were isolated from a very limited number of environments. Further analyses involving mobile elements of the different species belonging to the *Bifidobacteriaceae* family reveal varying percentages of such mobile elements (calculated as a proportion of the total number of genes within these genomes), ranging from 0.07% for *Bifidobacterium indicum* LMG 11587 to 5.02% for *B. hapali* DSM 100202. Furthermore, the overall *Bifidobacterium* genus contains a percentage of 1.5% mobile elements, while non-bifidobacterial species reveal a percentage of 0.9%, highlighting an approximate mobile element ratio of 2.1 (obtained by dividing the average contents of the predicted mobile elements of bifidobacterial (sub) species with that of the non-bifidobacterial taxa belonging to the *Bifidobacteriaceae* family). Thus, the larger abundance of predicted mobile elements in bifidobacterial genomes reflects the above mentioned increased genomic variability of these strains. Interestingly, the number of tRNA genes in bifidobacteria ranges from 40 for *Bifidobacterium thermacidophilum* subsp. *porcinum* LMG 21689 to 79 for *Bifidobacterium longum* subsp. *infantis* ATCC 15697, while in other members of the *Bifidobacteriaceae* the tRNA abundance seems to be much less variable, ranging from 45 to 48 (Table 1). Nevertheless, the *Bifidobacteriaceae* strains that do not belong to the *Bifidobacterium* genus possess at least one tRNA gene for each of the 20 amino acids (Additional file 1: Table S1). Additionally, a deeper screening of the anticodon sequences for each (sub) species does not display major differences, except for a lower abundance of the anticodon GGG in Proline tRNA of the *Bifidobacteriaceae* strains that do not belong to the *Bifidobacterium* genus (Additional file 1: Table S1). Consequently, it may be argued that the lower number of tRNA genes among certain members of the *Bifidobacteriaceae* family is not associated with a simplification of the codon usage of these strains. Furthermore, while the bifidobacterial genomes contain between one and six rRNA loci, with an average of 3.2 per genome [2], the genomes of the *Aeriscardovia*, *Alloscardovia*, *Bombiscardovia*, *Gardnerella*, *Neoscardovia*, *Parascardovia*, *Pseudoscardovia* and *Scardovia* genera exhibit a lower average number of rRNA loci, i.e., 2.6, which is consistent with the less extensive ORFome and tRNA arsenal identified in the corresponding genomes (Table 2).

Furthermore, in silico analyses of the 12 genomes of the *Bifidobacteriaceae* family based on the Virulence Factor Database (VFDB) [53], did not reveal the occurrence of any virulence genetic determinants. Such results confirm previously reported findings for the genome of *B. dentium* Bd1 [23].

### Pan-genome, core genome and unique genes of the *Bifidobacteriaceae* family

**Fig. 1:**
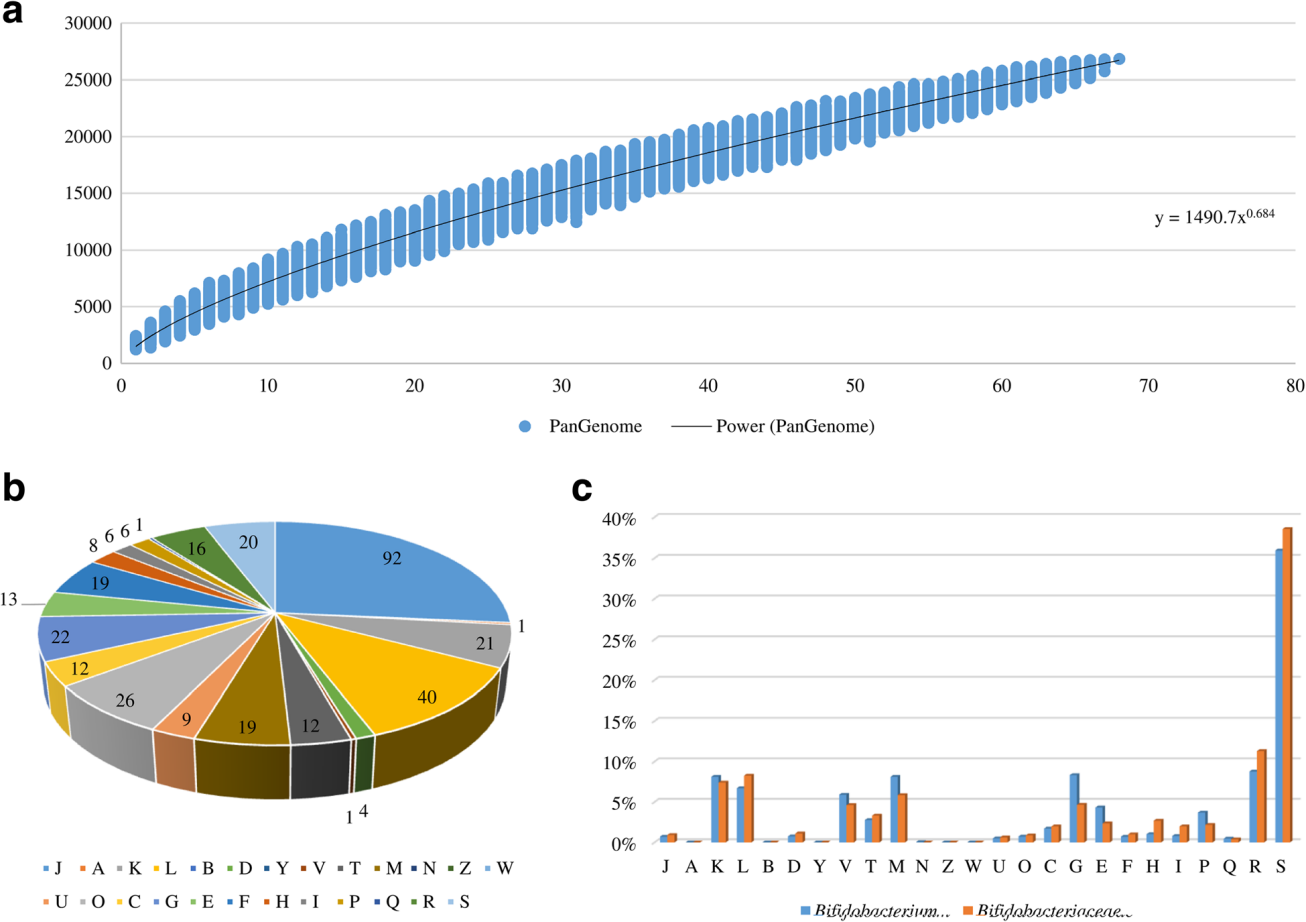
Pan-genome and functional classification of genes of the *Bifidobacteriaceae* family. Panel **a** shows the pan-genome represented as variations of the sizes of the resulting gene pool upon sequential addition of the 67 *Bifidobacteriaceae* genomes. The x axis represents the number of included genomes, whereas the y axis represents the number of genes in the generated pan-genome. Panel **b** exhibits the number of core BaeCOGs associated with the predicted EggNOG classification. Panel **c** displays the percentages of TUGs associated with functional categories as classified through the EggNOG database between bifidobacterial and members of the *Bifidobacteriaceae* family that do not belong to the *Bifidobacterium* genus. COG families are identified by a one-letter abbreviation: A, RNA processing and modification; B, chromatin structure and dynamics; C, energy production and conversion; D, cell cycle control and mitosis; E, amino acid metabolism and transport; F, nucleotide metabolism and transport; G, carbohydrate metabolism and transport; H, coenzyme metabolism; I, lipid metabolism; J, translation; K, transcription; L, replication and repair; M, cell wall/membrane/envelope biogenesis; N, cell motility; O, post translational modification, protein turnover, and chaperone functions; P, inorganic ion transport and metabolism; Q, secondary structure; T, signal transduction; U, intracellular trafficking and secretion; Y, nuclear structure; V, defense mechanisms; Z, cytoskeleton; R, general functional prediction only; S, function unknown

Comparative genome analyses involving the 67 (sub) species belonging to the *Bifidobacteriaceae* family were performed to unveil the corresponding pan-genome, core genome and unique genes of this bacterial family. All genomes were subjected to identical ORFs finding and annotation protocols [31] in order to generate comparable data sets for each *Bifidobacteriaceae* taxa. A total of 25,744 BaeCOGs (*Bifidobacteriaceae*-specific clusters of orthologous genes) were identified in the 67 *Bifidobacteriaceae* (sub) species, of which 8359 had members present in at least two genomes. The pan-genome size, when plotted versus the number of included genomes, clearly shows that the power trend line has yet to reach a plateau (Fig. 1). Actually, the number of new genes discovered by sequential addition of genome sequences was reduced from 839 to 636 BaeCOGs in the first three genomes additions to a number that ranged from 274 to 272 BaeCOGs in the final three additions, demonstrating the existence of an open pan-genome within *Bifidobacteriaceae* family. This finding suggests that more members of the *Bifidobacteriaceae* family have yet to be identified, especially members of the family that do not belong to the *Bifidobacterium* genus, as these remain poorly characterized in various environments compared to the currently recognized bifidobacterial (sub)species.

Pan-genome analysis of the *Bifidobacteriaceae* family allowed the identification of 353 COGs shared by all 67 (sub) species, representing the core genome of currently sequenced *Bifidobacteriaceae* representatives (core BaeCOGs). An examination of the functional annotation of Core BaeCOGs employing the eggNOG database [45] shows that the most conserved core genes specify housekeeping functions such as replication, transcription and translation, or functions related to adaptation such as carbohydrate, nucleotide and amino acid metabolism as well as cell envelope biogenesis (Fig. 1).

The pan-genome analysis also allowed the identification of truly unique genes (TUGs) of the *Bifidobacteriaceae* family, i.e., those genes that are presented in one particular strain yet absent in any of the other examined representative of the *Bifidobacteriaceae* family. The number of TUGs range from 42 for *B. indicum* LMG 11587 to 585 for *Bifidobacterium cuniculi* LMG 10738. EggNOG analysis showed that the majority of TUGs (59%) have no functional annotation (Additional file 1: Table S2). Nevertheless, taking into account the classified genes through the eggNOG analysis excluding the hypothetical and no-function genes, the highest number of genes fall in carbohydrate metabolism and cell envelope biogenesis together with replication and transcription. As mentioned above, these are the same four categories identified as containing the highest numbers of core BaeCOGs. Interestingly, the functional annotation of TUGs revealed that bifidobacterial genomes exhibit a higher abundance of TUGs involved in carbohydrate metabolism and cell envelope biogenesis compared to those of other members of the *Bifidobacteriaceae* family, reflecting a 38% and 78% of additional TUGs based on the average numbers between groups, respectively [2, 54] (Fig. 1 and Additional file 1: Table S2). These results are consistent with previous genomic and functional analyses based on the reference strains for each (sub) species of the *Bifidobacterium* genus indicating that bifidobacteria are under strong selective pressure to acquire and retain accessory genes for carbohydrate utilization in order to be competitive in the specific ecological niches in which they reside [2, 54].

### Phylogenomic analyses of members of the *Bifidobacteriaceae* family

**Fig. 2:**
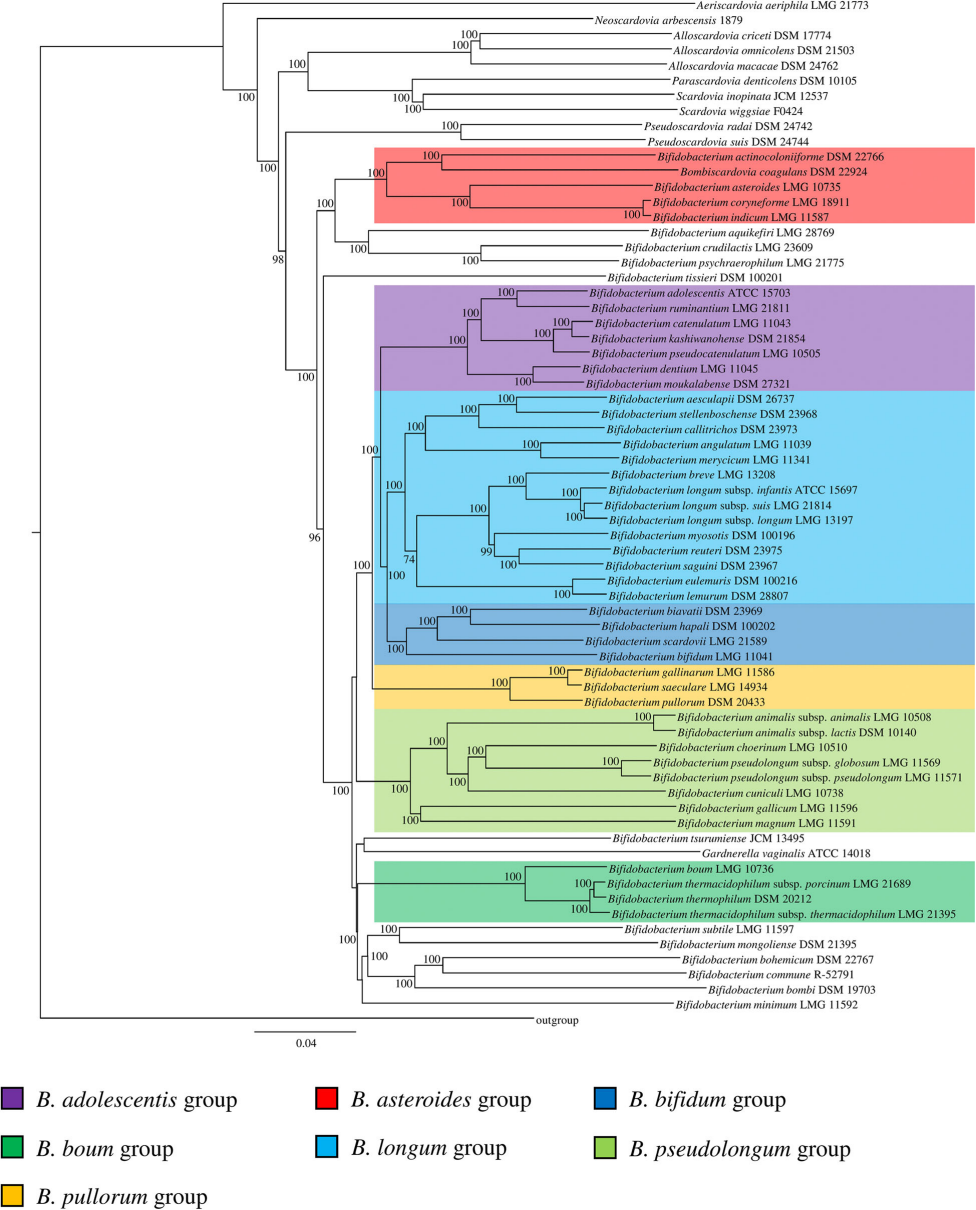
Supertree of the *Bifidobacteriaceae* family based on the concatenation of the amino acid sequences deduced from 314 core genes. Bootstrap values higher than 70 are marked near the respective nodes. Bifidobacterial groups are highlighted in different colors

The availability of genome sequences for each member of the *Bifidobacteriaceae* family allowed an in-depth analysis of the evolutionary development followed by each member of this extensive family. A phylogenetic supertree was constructed based on the concatenation of 314 protein sequences that represent the core BaeCOGs with the exclusion of paralogs identified in each genome (Fig. 2).

The generated phylogenetic supertree showed that ten strains of the *Bifidobacteriaceae* species that do not belong to the *Bifidobacterium* genus represent the deepest branches of this supertree, being separated from the 55 bifidobacterial (sub) species (Fig. 2). Consequently, the bifidobacterial species were positioned at the top of the supertree, reflecting their highest average gene ratio (1.24) as compared to the non-bifidobacterial members of the *Bifidobacteriaceae* family. Thus, these data clearly indicate that evolution of currently known bifidobacterial species involved a relatively limited number of ancestral gene loss incidences, yet an extensive number of gene acquisition events, corroborating previously published data [2]. Interestingly, two members of the *Bifidobacteriaceae* family that do not belong to the *Bifidobacterium* genus, i.e., *G. vaginalis* ATCC 14018 and *Bo. coagulans* DSM 22924, appear to possess a higher level of phylogenetic relatedness with bifidobacterial strains as compared to other, non-bifidobacterial members of the *Bifidobacteriaceae* family (Fig. 2). While *G. vaginalis* ATCC 14018 shares the same phylogenetic branch as that of *Bifidobacterium subtile* LMG 11597, *Bo. coagulans* DSM 22924 is positioned within the deepest branch of the *Bifidobacterium* genus, i.e., within the *B. asteroides* group [2, 10], exhibiting higher genome relatedness with *Bifidobacterium actinocoloniiforme* DSM 22766, which was also isolated from *Bombus*. In order to validate the branching of these two *Bifidobacteriaceae* strains, a phylogenetic tree based on the 16S rRNA genes was constructed, as well as a tree based on five housekeeping genes including *hsp*60, *rpo*B, *dna*J, *dna*G and *clp*C (Additional file 2: Figure S1). While *Bo. coagulans* DSM 22924 shares the same phylogenetic branch with *B. actinocoloniiforme* DSM 22766 in both trees, *G. vaginalis* ATCC 14018 occupies different positions within these phylogenetic trees. Nonetheless, the housekeeping-based tree confirmed the position of *G. vaginalis* ATCC 14018 within the *Bifidobacterium* genus, while in the 16S rRNA-based tree it is placed between bifidobacterial species and the other non-bifidobacterial species. These findings cast doubts on the correct taxonomical classification of *Bo. coagulans* DSM 22924, and reinforce the importance of a phylogenomic approach as a tool for taxonomic validation [55].

Furthermore, we investigated the occurrence of genes predicted to encode enzymes for anaerobic respiration in the pangenome of the members of the *Bifidobacteriaceae* as previously described for the genome of *B. asteroides* PRL2011 [10]. Such in silico analyses highlight the presence of a cytochrome *bd* oxidase-encoding complex in the genome of *Bo. coagulans* DSM 22924 (Additional file 1: Table S3), including the *cyd*A and *cyd*B, which code for structural subunit of the cytochrome, as well as *cyd*C and *cyd*D, encoding a transporter required for the cytochrome assembly [10]. Furthermore, these four genes were identified in 11 genomes of the *Bifidobacteriaceae* family, including six strains isolated from insects, i.e., *B. actinocoloniiforme* DSM 22766, *Bifidobacterium bohemicum* DSM 22767, *Bifidobacterium bombi* DSM 19703, *Bifidobacterium commune* R-52791, *Bifidobacterium coryneforme* LMG 18911 and *B. indicum* LMG 11587, highlighting a correlation between the presence of the cytochrome *bd* oxidase complex and the ecological niche of isolation (Additional file 1: Table S3).

Based on the *Bifidobacteriaceae* supertree reconstruction, five out of the six bifidobacterial species that were newly sequenced as part of this study are positioned within one of the previously identified bifidobacterial groups [24, 56]. Interestingly, four strains appear to belong to the *Bifidobacterium longum* group, i.e., *B. myosotis* DSM 100196, *B. reuteri* DSM 23975, *B. eulemuris* DSM 100216 and *B. lemurum* DSM 28807 (Fig. 2). This finding is in line with the particular ecological origin of these strains. In fact, each one of these four species was isolated from feces of monkeys, similar to the other two members that had previously been assigned to the *B. longum* group, i.e., *Bifidobacterium stellenboschense* DSM 23968 and *Bifidobacterium callitrichos* DSM 23973. These findings therefore highlight that the *B. longum* group includes bifidobacterial (sub) species isolated from humans and other related primates (Fig. 2). Furthermore, *B. hapali* DSM 100202 exhibits a genetic relatedness with *Bifidobacterium biavatii* DSM 23969, which belongs to the *B. bifidum* group [24]. Notably, *B. tissieri* DSM 100201 was shown to occupy a unique position within the *Bifidobacteriaceae* supertree. This observation was confirmed through an average nucleotide identity (ANI) analysis showing the highest value of 88.02% with the most related *Alloscardovia criceti* DSM 17774 strain. Notably, an ANI value below to 95% is assumed to be sufficient to classify that taxon as a distinct species [43]. *B. tissieri* DSM 100201 is therefore an interesting strain for further investigation due to its genetic divergence from other bifidobacterial taxa. In order to validate the species assignment for the other 10 sequenced members of the *Bifidobacteriaceae* family (Table 2) from a genomic prospective, the decoded genomes were subjected to ANI comparisons with the other 56 (sub) species that had been sequenced. The sequenced genomes of the 10 *Bifidobacteriaceae* strains showed ANI values below 95%, confirming their status as distinct species, with a relatively high value of 93.8% observed between *B. eulemuris* DSM 100216 and *B. lemurum* DSM 28807 (Additional file 1: Table S4).

Furthermore, the *Bifidobacteriaceae* supertree confirmed the phylogenetic relatedness of the recently decoded genome sequences of *B. commune* R-52791 and *Bifidobacterium aesculapii* DSM 26737, which occupy positions within the same branches with *B. bohemicum* DSM 22767 and *B. stellenboschense* DSM 23968, respectively [49, 57]. Such data further confirms the existence of a direct relatedness between the ecological origin of both strains, i.e., insects of the *Bombus* genus and monkeys of the *Callitrichidae* family.

### Enzymatic profiling and evolutionary development of the *Bifidobacteriaceae* family

A functional profiling analysis was performed to assess the presence of genes encompassing carbohydrate, amino acid and fatty acid degradation pathways in each (sub) species of the *Bifidobacteriaceae* family. Normalizing the obtained number of gene matches with the overall genetic arsenal (i.e. total amount of genes) of each strain, non-bifidobacterial strains retrieved percentages that were slightly lower compared to the bifidobacterial strains, i.e., 7%, 11% and 9%, respectively (Additional file 1: Table S5). Interestingly, all members of the *Bifidobacteriaceae* family possess the *Bifidobacterium* shunt pathway [6, 58], including the gene *xfp* that encodes the enzyme D-xylulose 5-phosphate phosphoketolase/D-fructose 6-phosphate phosphoketolase, expanding the notion of this signature metabolic trait of bifidobacteria to the whole family.

To further investigate the carbohydrate utilization abilities encoded by the genomes of the 67 *Bifidobacteriaceae* (sub) species, an enzyme classification toward glycans was performed. This enzyme classification was based on the Carbohydrate Active Enzymes (CAZy) database [44], which encompasses all currently known genetic determinants involved in the breakdown and utilization of carbohydrates, and revealed that the pan-genome of the *Bifidobacteriaceae* family includes 9742 genes predicted to encode carbohydrate-active enzymes, i.e., glycosyl hydrolases (GHs), glycosyl transferases (GTs), polysaccharide lyases (PLs), carbohydrate esterases (CEs) and carbohydrate binding modules (CBMs), present at 43.4%, 43.8%, 0.2% 5.9% and 12.6%, respectively. This very substantial number of retrieved enzymes reflects the findings of a previous analysis conducted on the 47 type-strains of the *Bifidobacterium* genus, where the glycan-breakdown potential of bifidobacteria in the mammalian gut was subjected to an extensive scrutiny [54].

Focusing on GH identification, 3989 genes were predicted on the analyzed genomes of 55 bifidobacterial (sub) species, while the remaining 12 *Bifidobacteriaceae* genomes contain 573 such genes. The *Bifidobacteriaceae* genomes specify a large arsenal of GH families, were GH13, GH3, GH43, GH23, GH32 and GH25 outnumber the other identified families (Additional file 1: Table S6). Despite the higher average amount of predicted GH-encoding genes for a given bifidobacterial genome, i.e., 72, as compared to that for a non-bifidobacterial member of the *Bifidobacteriaceae* family, i.e., 48, normalization of GH counts against the total amount of predicted genes provided similar GH indexes, i.e., 0.039 and 0.032, respectively (Additional file 1: Table S6). Nonetheless, genomes of the 20 strains that exhibit the highest GH index all belong to the *Bifidobacterium* genus, and are nearly all isolated from fecal samples of humans and monkeys, chief among them being *Bifidobacterium catenulatum* LMG 11043, *B. eulemuris* DSM 100216, *B. hapali* DSM 100202 and *B. biavatii* DSM 23969 (Table 1). These results suggest that bifidobacteria that reside in the primate/human gut have enjoyed a relatively high number of adaptive events related to carbohydrate metabolism to benefit from a wider source of different nutrients present in this particular environment. In contrast, *Bifidobacteriaceae* species that exhibit a relatively low GH index originate from a broad spectrum of environments, such as the gut of insects or other animals, and sewage (Table 1). Furthermore, genomes with a low GH index correspond with the smaller genomic complement of certain members of the *Bifidobacteriaceae* family, probably due to gene decay characteristic of those microbes that are considered harmful for human health, such as *G. vaginalis*, *A. omnicolens*, *S. inopinata* and *S. wiggsiae* [59].

**Fig. 3:**
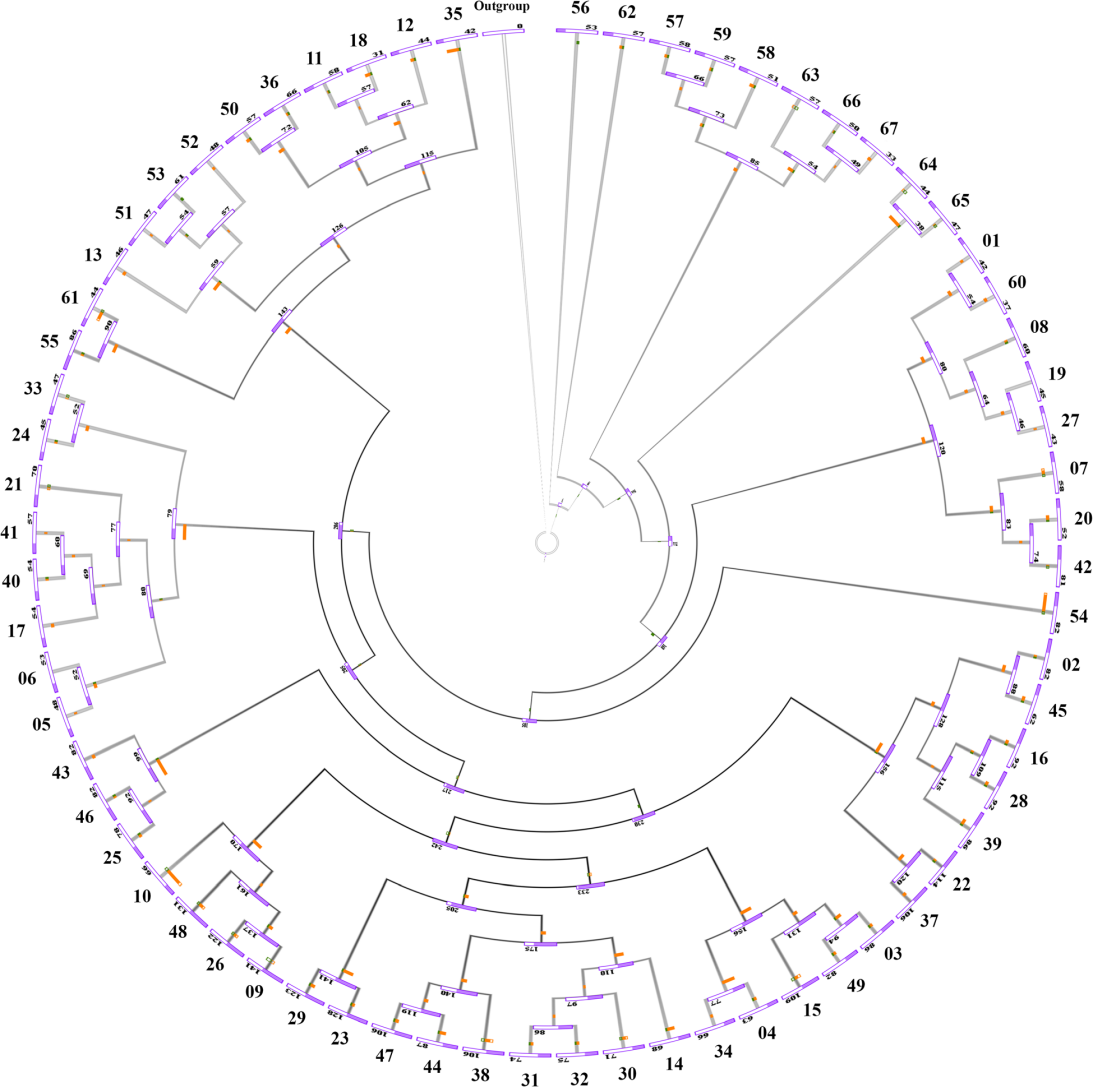
Analysis of evolutionary gain and loss events as based on predicted GHs within the *Bifidobacteriaceae* family. The image displays the core gene-based supertree of the *Bifidobacteriaceae* family, where each node reports the number of predicted GHs identified for each strain. Furthermore, gain and loss events are indicated by *green* and *orange bars* on the edge leading to each node, while the numbers placed at the *supertree leaves* represent the related bifidobacterial taxa presented in Table 1

While gene acquisition events that occur during evolution of microbial genomes support adaptation to new ecological niches, gene loss on the other hand contributes to genome simplification in order to preserve energy and biological compounds [59, 60]. Prediction of the complete *Bifidobacteriaceae* glycobiome content allowed us to estimate the acquisition and loss rates of genes encoding carbohydrate-active enzymes within this saccharolytic family. In order to depict gain and loss events of genes with a predicted function in carbohydrate metabolism, we collected the BaeCOGs that include GH-encoding genes obtained from the pan-genome analysis. The resulting 846 BaeCOGs were allocated among the *Bifidobacteriaceae* supertree showing that evolution of the current bifidobacterial (sub) species involved only a limited number of ancestral gene loss events, yet a substantial number of GH-encoding gene acquisitions (Fig. 3). Thus, genes encoding GHs appear to have been acquired early in the evolution of bifidobacteria, followed by a simplification of the GH-associated gene arsenal that has resulted in or followed specialization toward those ecological niches in which current bifidobacterial species have been identified. Interestingly, members of the *B. bifidum* group possess the highest number of GH-encoding gene acquisitions compared to those of other groups, probably in order to expand its metabolic ability towards different carbon sources present in the host, similar to what was mentioned above regarding GH index discrepancies (Fig. 3 and Table 1). Taking into account only those BaeCOGs that include members of GH families known to be involved in host-glycan degradation, i.e., GH20, GH29, GH33, GH38, GH95, GH101, GH112, GH125 and GH129 [54], the *B. bifidum* group, once again, displays the highest number of GH-encoding gene acquisitions (up to seven BaeCOGs). In this context *B. bifidum* exhibits the highest host-glycan degradation BaeCOGs acquisition number of the *Bifidobacteriaceae* family, thereby highlighting the capability of this bifidobacterial species to feed on host-glycan [25, 61, 62].

## Conclusions

A lot of effort has been invested in the dissection and characterization of the genomic content from different members of the *Bifidobacterium* genus [2, 11, 24–28]. In contrast, very little is known about the genomics of other members of the *Bifidobacteriaceae* family, which include apart from the *Bifidobacterium* genus eight additional genera. In this study, genome sequencing allowed us to explore the genome content of known members across the *Bifidobacteriaceae* family, as represented by 67 (sub) species, and to scrutinize the phylogenetic relatedness between each taxon belonging to this family. Bifidobacteria exhibit a higher number of genes per genome compared to other members of the *Bifidobacteriaceae* family, perhaps reflecting an increased competitiveness based on a broad spectrum of ecological niches from which they were isolated. The more complex genome content of bifidobacteria is also reflected by the capability of these microorganisms to degrade multiple carbon sources [54, 63]. Such findings were further validated in this study by the analyses of the pan-genome of the members of the *Bifidobacteriaceae* family, which highlights the occurrence of a higher abundance of TUGs in bifidobacterial genomes dedicated to the carbohydrate metabolism and cell envelope biogenesis, when compared to such numbers for other members of the *Bifidobacteriaceae* family. Enzymatic gene profiling revealed that the 20 strains that showed the highest GH indexes belong to bifidobacterial strains that are nearly all isolated from humans or monkeys. Moreover, a gene gain/loss analysis shows that members of the *Bifidobacterium* genus isolated from such primates possess the highest number of GH-encoding gene acquisitions, probably in order to expand their ability to metabolize different carbon sources. These results highlight a relatively large number of adaptive events related to carbohydrate metabolism among members of the *Bifidobacterium* genus that reside in omnivorous organisms that consume a wide variety of nutrients.

Furthermore, our phylogenomic analysis revealed possible taxonomic inconsistencies in the classification of *G. vaginalis* and *Bo. coagulans*, which displayed a close phylogenetic relatedness with other bifidobacterial strains, i.e., *B. subtile* LMG 11597 and *B. actinocoloniiforme* DSM 22766. Such findings further corroborate the strengths of genome-based analyses as an essential approach to be incorporated in phylogenome-based taxonomic studies.

## Additional files

Additional file 1: **Tables S1**, **S2**, **S3**, **S4**, **S5** and **S6**. (XLSX 3628 kb)

Additional file 2: Figure S1. Phylogenetic trees of the *Bifidobacteriaceae* family. Panel a display the 16S rRNA gene-based tree of the current recognized (sub) species of the family. Panel b shows the phylogenetic tree based on the concatenation of the amino acid sequences of five housekeeping genes including *hsp*60, *rpo*B, *dna*J, *dna*G and *clp*C. For each tree, bootstrap values higher than 70 are marked near the respective nodes. (TIFF 2260 kb)

## Abbreviations

ANI: Average nucleotide identity
BaeCOGs: Bifidobacteriaceae-specific clusters of orthologous groups
CAZy: Carbohydrate active enzymes
CBMs: Carbohydrate binding modules
CEs: Carbohydrate esterases
GF: Gene family
GHs: Glycosyl hydrolases
GIT: Gastrointestinal tract
GTs: Glycosyl transferases
HMM: Hidden Markov Model
MRS: Man-Rogosa-Sharpe
NCBI: National Center for Biotechnology Information
ORFs: Open reading frames
PLs: Polysaccharide lyases
TUGs: Truly unique genes
