# Supplementary figures and images for: Comparative genomic and phylogenomic analyses of the Bifidobacteriaceae family
Source: BMC Genomics. 2017 Aug 1;18:568. doi: 10.1186/s12864-017-3955-4 (PMC5540593; doi:10.1186/s12864-017-3955-4)

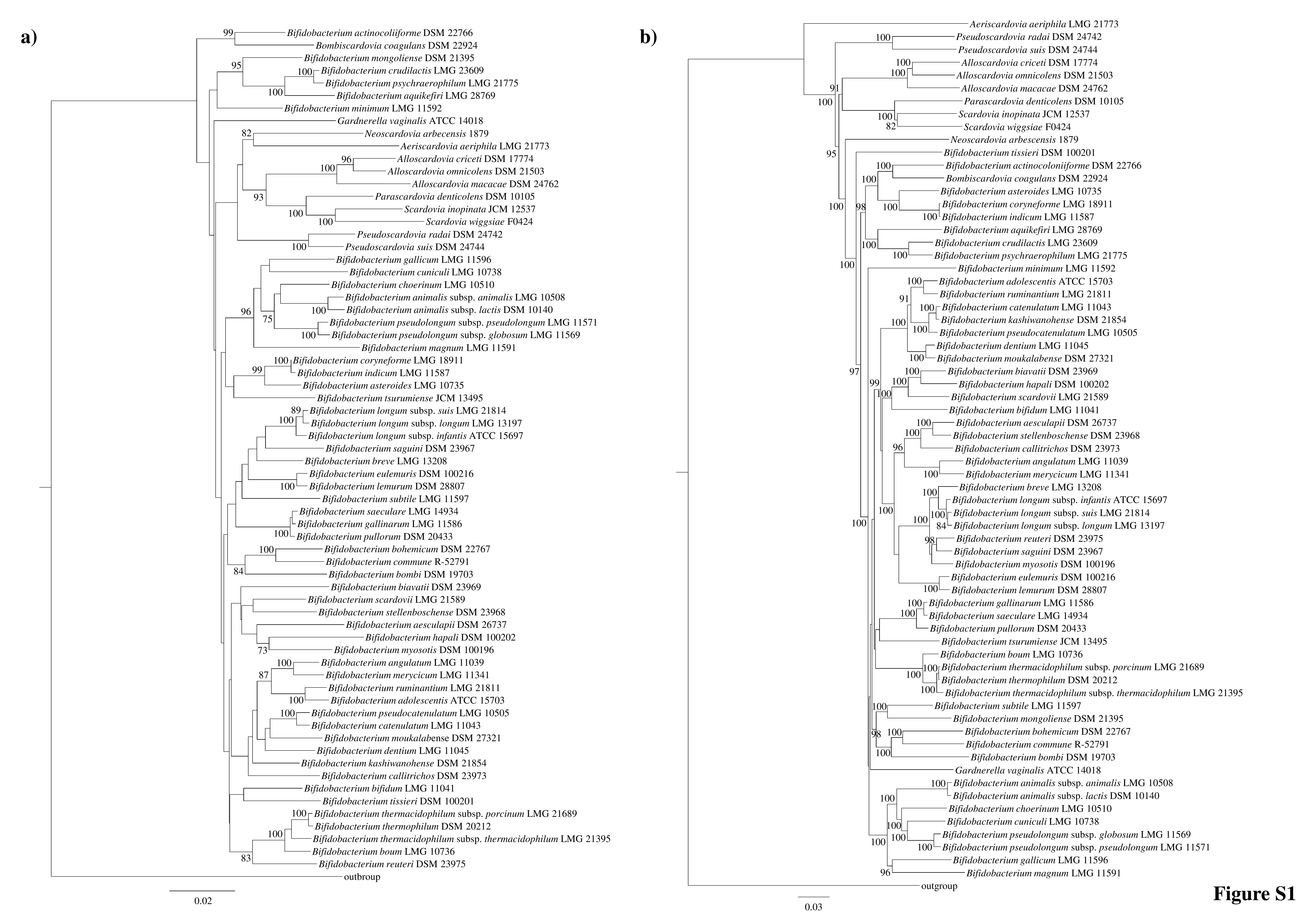

Supplement: Supplementary file 2 — Phylogenetic trees of the Bifidobacteriaceae family. Panel a display the 16S rRNA gene-based tree of the current recognized (sub) species of the family. Panel b shows the phylogenetic tree based on the concatenation of the amino acid sequences of five housekeeping genes including hsp60, rpoB, dnaJ, dnaG and clpC. For each tree, bootstrap values higher than 70 are marked near the respective nodes. (TIFF 2260 kb) [file 12864_2017_3955_MOESM2_ESM.tif]
